# Artificial food dyes are toxic: Neurobehavioral implications in children

**DOI:** 10.1016/j.bas.2024.102869

**Published:** 2023-07-11

**Authors:** Gokul Sudhakaran

**Affiliations:** Center for Global Health Research, Saveetha Medical College and Hospital, Saveetha Institute of Medical and Technical Sciences (SIMATS), Thandalam, Tamil Nadu, India

**Keywords:** Food dyes, Synthetic dyes, Behavior

## Abstract

Emerging research highlights the potential neurobehavioral impacts of synthetic food dyes on children, prompting a reevaluation of their safety and regulatory standards. This letter discusses recent findings that associate synthetic food dyes with adverse behavioral outcomes, such as hyperactivity, particularly in children with or without identified behavioral disorders. It calls for updated regulatory guidelines that reflect current research, advocating for protecting children's behavioral health.

Dear Editor,

Recent evaluations of synthetic food dyes raise significant concerns about their safety, particularly regarding children's neurobehavioral health (Haridevamuthu et al., 2024). A comprehensive review conducted by the California Environmental Protection Agency's Office of Environmental Health Hazard Assessment (OEHHA) and detailed in a report released by the state of California in April 2021 has illuminated the longstanding suspicions regarding the impact of these dyes on children's behavior, including hyperactivity and other neurobehavioral issues (Office of Environmental Health Hazard Assessment and O, 2021).

The review encompassed human and animal studies, revealing that synthetic food dyes might lead to neurobehavioral effects manifesting during development or later in life. This is particularly concerning given the widespread consumption of these dyes across various food products aimed at children. Of the clinical trials reviewed, a significant portion reported adverse outcomes related to hyperactivity or inattention, underscoring the potential risk posed by these substances.

Moreover, the Acceptable Daily Intakes (ADIs) for synthetic food dyes, established between the 1960s and 1980s based on general toxicology studies, do not account for these neurobehavioral effects. Comparisons with newer studies suggest that current ADIs may not adequately protect children from the observed behavioral effects, indicating a pressing need for regulatory reassessment (Miller et al., 2022).

This discussion is supported by the findings of UC Berkeley andUC Davis, which confirm the link between synthetic food dye consumption and neurobehavioral issues in some children (California Environmental Protection Agency). The report highlights a discrepancy between the FDA's ADIs for artificial food dyes and the most current research, suggesting that existing guidelines may not sufficiently safeguard children's behavioral health.

Given the increasing prevalence of behavioral disorders among children and the role that environmental factors, including dietary additives like synthetic food dyes, may play in exacerbating these conditions, it is imperative to prioritize the health and well-being of our children. This includes revisiting and potentially revising the regulatory standards governing the use of synthetic food dyes in food products, ensuring they reflect the latest scientific understanding and effectively protect against adverse neurobehavioral outcomes.

## Declaration of competing interest

The authors declare that they have no known competing financial interests or personal relationships that could have appeared to influence the work reported in this paper.
